# Mitochondria: from powerhouses of cells to hubs in antitumor immunity

**DOI:** 10.3389/fimmu.2026.1852236

**Published:** 2026-06-09

**Authors:** Xiaofeng Li, Yanfeng Shi, Yan Zhao, Gengjun Zhu, Lifang Jin

**Affiliations:** 1 Department of Oncology and Hematology, The Second Hospital of Jilin University, Changchun, China; 2 Medical Records Department, The Affiliated Hospital of Changchun University of Chinese Medicine, Changchun, China; 3 Central Laboratory, The Second Hospital of Jilin University, Changchun, China

**Keywords:** antitumor immunity, CAR-T, metabolic reprogramming, mitochondria, tumor microenvironment

## Abstract

While immune checkpoint inhibitors and chimeric antigen receptor T-cell (CAR-T) therapies constitute the cornerstone of current immunotherapy, their efficacy is often limited by, most notably, the immunosuppressive tumor microenvironment. Recently, mitochondria are recognized as pivotal metabolic-immune hubs that critically support tumor progression, metastasis, and immune evasion. However, this insight has not yet translated into a clear understanding of the underlying mechanisms or their therapeutic potential. This review summarizes the role of mitochondria in cellular metabolic regulation, with a focus on mitochondrial−mediated metabolic reprogramming in cancer and immune cells within the tumor microenvironment. We then discuss therapeutic opportunities to potentiate antitumor immunity by targeting mitochondrial reprogramming in cancer and CAR-T cells. Finally, we offer a forward-looking perspective on emerging mitochondria−targeted strategies, such as mitochondrial vaccines, precise mtDNA editing, and engineered mitochondrial transplantation.

## 1 Introduction

Immune checkpoint blockade and adoptive cell therapies like chimeric antigen receptor T-cell (CAR-T) have launched a new era of cancer immunotherapy (1). While CAR-T therapy has achieved great success in hematologic malignancies, its effectiveness in solid tumors is still limited (2–4), and a significant proportion of patients fail to achieve a durable benefit from immune checkpoint inhibitors (ICIs) (5). The precise mechanisms underlying the limited efficacy of immunotherapy remain incompletely understood. Accumulating evidence highlights the immunosuppressive tumor microenvironment (TME) as a critical factor impairing the tumor-killing capacity of both cytotoxic T lymphocytes (CTLs) and adoptive CAR-T cells (6–9). Critically, the immunosuppressive TME is underpinned by profound metabolic reprogramming within tumor and stromal cells, which fuels a milieu characterized by nutrient depletion, acidosis, hypoxia, and chronic inflammation (10–13). Hence, disrupting the aberrant metabolic reprogramming within the TME has emerged as a promising strategic direction in cancer immunotherapy.

Recent studies have established the central role of mitochondria in metabolic reprogramming in both cancer and immune cells, far beyond their canonical function as cellular powerhouses (14). Notably, this reprogramming drives a multifaceted remodeling of the immunosuppressive tumor microenvironment. Mitochondria fitness plays a central role in regulating immune cell differentiation, effector function, and memory formation (15–18). A key mechanism underlying this regulation is epigenetic remodeling, with core tricarboxylic acid (TCA) cycle intermediates serving as essential cofactors or substrates (19–21). Beyond these metabolites, dysfunctional mitochondria themselves can be transferred from tumor to immune cells via tunneling nanotubes (TNTs) or extracellular vesicles, directly disrupting the recipient cells’ metabolic fitness and antitumor capacity (22, 23). Furthermore, in tumor cells, metabolic reprogramming manifests as the Warburg effect. This aerobic glycolysis not only supports anabolism but also fuels immune escape through lactate, which acidifies the microenvironment and induces immunosuppressive lactylation modifications (24–26).

**Figure 1:**
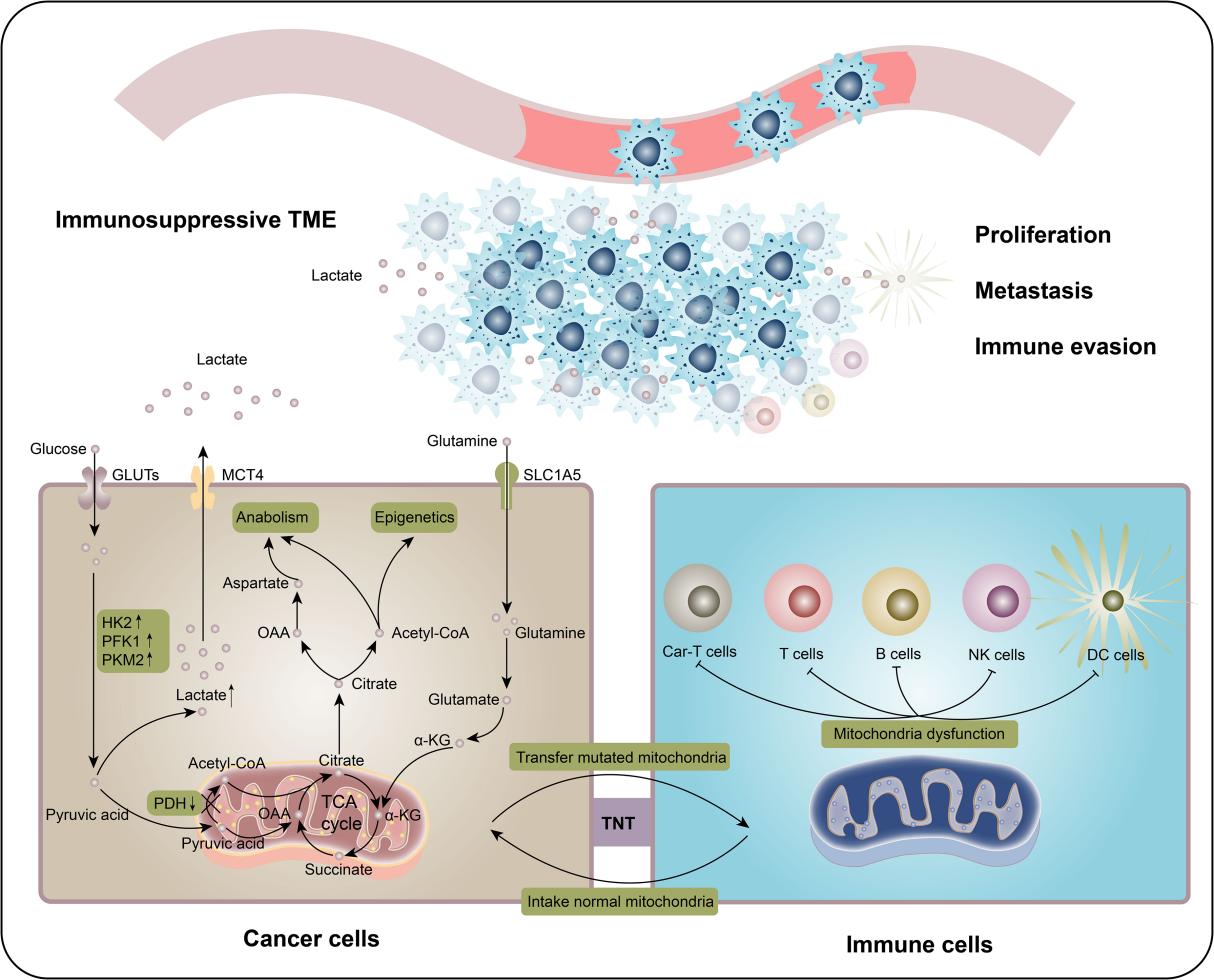
Mitochondrial metabolism reprogramming in cancer cells and immune cells. In cancer cells, the primary energy supply arises from aerobic glycolysis, driven by the activation of glycolytic key enzymes—HK2, PFK1, and PKM2—along with the downregulation of pyruvate dehydrogenase, a critical enzyme in the mitochondrial TCA cycle. Additionally, α-KG generated via glutamine anaplerosis and OAA derived from pyruvate carboxylation serve as important carbon sources fueling the TCA cycle. Citrate exported from mitochondria into the cytosol further supports anabolic reactions and epigenetic regulation. Moreover, tumor cells can uptake functional mitochondria from immune cells within the TME while transferring damaged mitochondria to immune cells, thereby suppressing antitumor immunity. Collectively, these mechanisms sustain an immunosuppressive microenvironment, facilitating tumor cell proliferation, metastasis, and immune evasion. TME, tumor microenvironment; AOO, Oxalacetate; HK2, hexokinase 2; PFK1, phosphofructokinase-1; PKM2, pyruvate kinase M2; PDH, pyruvate dehydrogenase complex; TCA, tricarboxylic acid cycle; DC, dendritic cell; Car-T, chimeric antigen receptor T-cell.

This review provides a comprehensive discussion of the central role of mitochondria in cellular metabolic regulation, followed by an in-depth exploration of mitochondrial-mediated metabolic reprogramming in both cancer cells and immune cells within the tumor microenvironment, as shown in **Figure 1**. The review further examines the implications of interventions targeting such reprogramming for antitumor immunity. Finally, it offers a forward-looking perspective on the development of mitochondrial vaccines, the advancement of precise mitochondrial DNA editing techniques, and the progress in engineered mitochondrial transplantation.

## 2 Mitochondria function as central hub for cellular metabolism

Research over the past few years has revealed that mitochondria are far more than ATP-generating organelles; they are now viewed as integrative metabolic hubs (27, 28). The TCA cycle acts as a central platform within this hub, enabling it to coordinate cellular anabolism and catabolism across diverse pathways, including glucose, lipid, amino acid, and one−carbon metabolism (14). Thus, elucidating the mechanisms underlying this integrative function is fundamental to understanding their roles in the tumor microenvironment.

### 2.1 Mitochondria and glucose metabolism

For highly proliferative cells such as cancer cells, glucose is the predominant nutrient supporting anabolic growth (29). In normal cellular metabolism, glucose under hypoxic conditions is metabolized via glycolysis to produce lactate and ATP. In contrast, under aerobic conditions, glycolytically-derived pyruvate enters the mitochondria for oxidation, where it is decarboxylated to acetyl-CoA. Then, the acetyl-CoA undergoes complete oxidation via the TCA cycle and the subsequent oxidative phosphorylation to generate the bulk of cellular ATP (28).

Beyond their central role in oxidative phosphorylation, mitochondria actively govern the entire glucose utilization pathway—from cellular uptake and glycolytic flux to terminal oxidation. On one hand, mitochondrial energy metabolites—such as ATP, NADH, citrate and acetyl-CoA—directly allosterically modulate major glycolysis rate-limiting enzymes including hexokinase-2 (HK2), phosphofructokinase-1 (PFK1) and pyruvate kinase M2 (PKM2) (30). On the other hand, mitochondrial signaling molecules—including ROS, and metabolites like succinate and α-KG—act through pathways like HIF−1α, AMPK, and mTOR to regulate cellular metabolism, impacting glucose uptake, glycolytic flux, and mitochondrial oxidative capacity (27). Therefore, mitochondria serve as an integrative hub for glucose metabolism, sensing the cellular energy status and orchestrating the entire metabolic cascade—from glucose uptake and glycolysis to aerobic oxidation—to ensure cellular energy homeostasis.

### 2.2 Mitochondria and lipid metabolism

Fatty acids are crucial source of cellular energy in the body, capable of generating a substantial amount of ATP through oxidative metabolism (31). The energy yield from 1 mol of fatty acids (106 ATP) is significantly greater than that from 1 mol of glucose (36 ATP) (32). The mitochondria are the primary sites for fatty acid oxidation. In the cytosol, fatty acids are activated to fatty acyl-CoA, which is then transported into the mitochondria via carnitine palmitoyltransferase I (CPT1). Within mitochondria, fatty acyl-CoA is catabolized through β-oxidation, a cyclic process comprising oxidation, hydration, dehydrogenation, and thiolytic cleavage, yielding a significant amount of acetyl-CoA (33). These acetyl-CoA either entered the tricarboxylic acid (TCA) cycle for complete oxidation or was utilized for the synthesis of other metabolic intermediates within the mitochondria (34). The mitochondrion functions as a primary gatekeeper of fatty acid oxidation by controlling its rate-limiting entry step, thus dynamically matching fuel supply with cellular energy and biosynthetic demands to preserve homeostasis.

Mitochondria also play crucial roles in lipid synthesis. The mitochondrion supplies acetyl-CoA—the fundamental two-carbon building block—as the essential substrate for lipogenesis in the endoplasmic reticulum (35). To export mitochondrial acetyl-CoA for cytosolic lipogenesis, cells employ the classic citrate-pyruvate cycle. In this cycle, acetyl-CoA condenses with oxaloacetate to form citrate, which is exported to the cytosol via the mitochondrial citrate carrier. Cytosolic ATP-citrate lyase (ACLY) then cleaves citrate to regenerate acetyl-CoA for fatty acid synthesis and oxaloacetate, which is reduced to malate and decarboxylated to pyruvate before re-entering the mitochondrion, thus completing the cycle (36). Notably, mitochondrial metabolites—including citrate, isocitrate, and acetyl-CoA—serve as critical regulatory signals for fatty acid synthesis (37). Apart from their role as a metabolic supplier, mitochondria further modulate lipogenesis through AMPK signaling pathways (38). Consequently, mitochondria act as central regulatory hubs that coordinate both the synthesis and catabolism of fatty acids.

### 2.3 Mitochondria and amino acid metabolism

Amino acids are the basic units of cellular proteins, and the homeostasis of their metabolism is critical for cell survival. The mitochondria act as the central hub for integrating and regulating amino acid metabolism. It serves not only as the primary site for catabolizing amino acids but also as a central coordinator of overall amino acid metabolism, which is achieved by monitoring cellular energy and nutrient levels, and accordingly regulating the flow of key metabolic intermediates (39, 40).

In amino acid catabolism, the carbon skeletons are processed to generate α-keto acids via reactions like oxidative deamination. These α-keto acids then enter the mitochondrial tricarboxylic acid (TCA) cycle for oxidation and energy production. Specifically, glutamate, a central amino group carrier, is oxidatively deaminated within the mitochondrial matrix by the enzyme glutamate dehydrogenase (41). This reaction yields α-KG and ammonia, thereby linking amino acid nitrogen disposal to central carbon metabolism. Concurrently, the mitochondrion plays an indispensable role in nitrogen detoxification. The initial and rate-limiting steps of the urea cycle—the synthesis of carbamoyl phosphate and the formation of citrulline—occur within the mitochondrial matrix (42). In the urea cycle, this toxic ammonia is captured by combining with other metabolites to form citrulline, a non-toxic molecule that is then transported to the cytosol for further processing.

Conversely, mitochondria also supply precursors for amino acid synthesis. Key metabolites—including the TCA cycle intermediates oxaloacetate and α-ketoglutarate, as well as pyruvate—can be exported from the mitochondria. They then serve as carbon backbones for the biosynthesis of aspartate, glutamate, and alanine, respectively, through transamination reactions (43). In summary, the mitochondrion acts as a central integrator of amino acid metabolism. It maintains cellular amino acid homeostasis and metabolic adaptability by modulating their catabolism, anabolism, and interconversion in response to energy and nutrient signals.

### 2.4 Mitochondrion and one-carbon unit metabolism

One-carbon units are single-carbon groups (such as methyl, methylene, methenyl, and formyl) generated during the catabolism of certain amino acids (44). Their primary carriers are folate and methionine, and they play critical roles in nucleic acid synthesis, methylation modifications, and, consequently, in regulating cell proliferation and gene expression (45). Mitochondria maintain an independent folate pool and possess specific transporters (46). The integrity of mitochondrial function is a prerequisite for maintaining the proper distribution and stable supply of the cellular folate pool. Within mitochondria, serine hydroxymethyltransferase (SHMT2) converts serine to glycine, simultaneously generating 5,10-methylenetetrahydrofolate (5,10-CH_2_-THF), which represents the major source of one-carbon units (47). Mitochondria strategically direct the fate of 5,10-CH_2_-THF based on cellular energy status. Under high energy demand, the flux is favored toward the glycine cleavage system (GCS) to generate NADH, thereby supporting the respiratory chain (48). Conversely, during periods of high anabolic demand, such as rapid cell proliferation, the unit is oxidized to formate, which is exported to the cytosol for nucleotide synthesis (49).

The methionine cycle serves as another crucial pathway for methyl group transfer (50). Methionine, containing an S-methyl group, reacts with ATP catalyzed by methionine adenosyltransferase (MAT) to form S-adenosylmethionine (SAM). SAM is the most important universal methyl donor in the cells and is often referred to as “active methionine” (51, 52). It functions dually: 1) it participates in the epigenetic regulation of gene expression by providing methyl groups for DNA and histone methylation, and 2) it provides aminopropyl groups for polyamine synthesis, which is integral to intracellular proliferation signaling (53). Following methyl transfer, SAM is demethylated to S-adenosylhomocysteine (SAH), which is then hydrolyzed to homocysteine (52). Homocysteine is subsequently remethylated back to methionine in a reaction catalyzed by methionine synthase, which utilizes N5-methyltetrahydrofolate (5-CH_3_-THF) as the methyl donor, thereby closing the methionine cycle (50). Mitochondria influence this cycle both by contributing to folate metabolism and by sensing intracellular SAM levels (54). This sensing occurs, in part, through SAM-dependent lipoic acid synthesis, as lipoic acid is an essential cofactor for key mitochondrial enzymes, including pyruvate dehydrogenase (PDH) and α-ketoglutarate dehydrogenase, thereby linking SAM availability directly to core mitochondrial respiration (54).

### 2.5 Mitochondria and redox homeostasis

Mitochondria are both the primary source of reactive oxygen species (ROS) and the cellular organelle richest in antioxidant proteins (55). The mitochondrial electron transport chain (particularly complexes I and III) serves as the major site for the generation of physiological ROS (56). During electron transfer, a small fraction of electrons “leak” and combine with molecular oxygen to form the ROS. At physiological levels, ROS—especially hydrogen peroxide (H_2_O_2_)—function as crucial second messengers, regulating key cellular pathways such as proliferation, differentiation, autophagy, hypoxia adaptation (via HIF-1α stabilization), and inflammatory responses (57).

However, excessive ROS production leads to oxidative damage of macromolecules including lipids, proteins, and DNA, thereby mediating various forms of programmed cell death (57). To manage this intrinsic ROS generation and prevent oxidative injury, mitochondria possess the most concentrated and specialized antioxidant defense network. Manganese superoxide dismutase (Mn-SOD) specifically catalyzes the conversion of superoxide anions into hydrogen peroxide (58). Subsequently, a system comprising glutathione peroxidases (GPXs), peroxiredoxins (PRDXs), catalase, the thioredoxin system, and glutathione (GSH) collaborates to catalyze the reduction of hydrogen peroxide to water, thereby mitigating mitochondrial oxidative stress (57, 59). Beyond this enzymatic defense, mitochondria employ dynamic quality control mechanisms—including fission, fusion, and mitophagy—to segregate, repair, or selectively remove severely damaged mitochondria (60). This process reduces ROS production at its source and is essential for maintaining a healthy mitochondrial network and overall cellular redox homeostasis.

**Figure 2:**
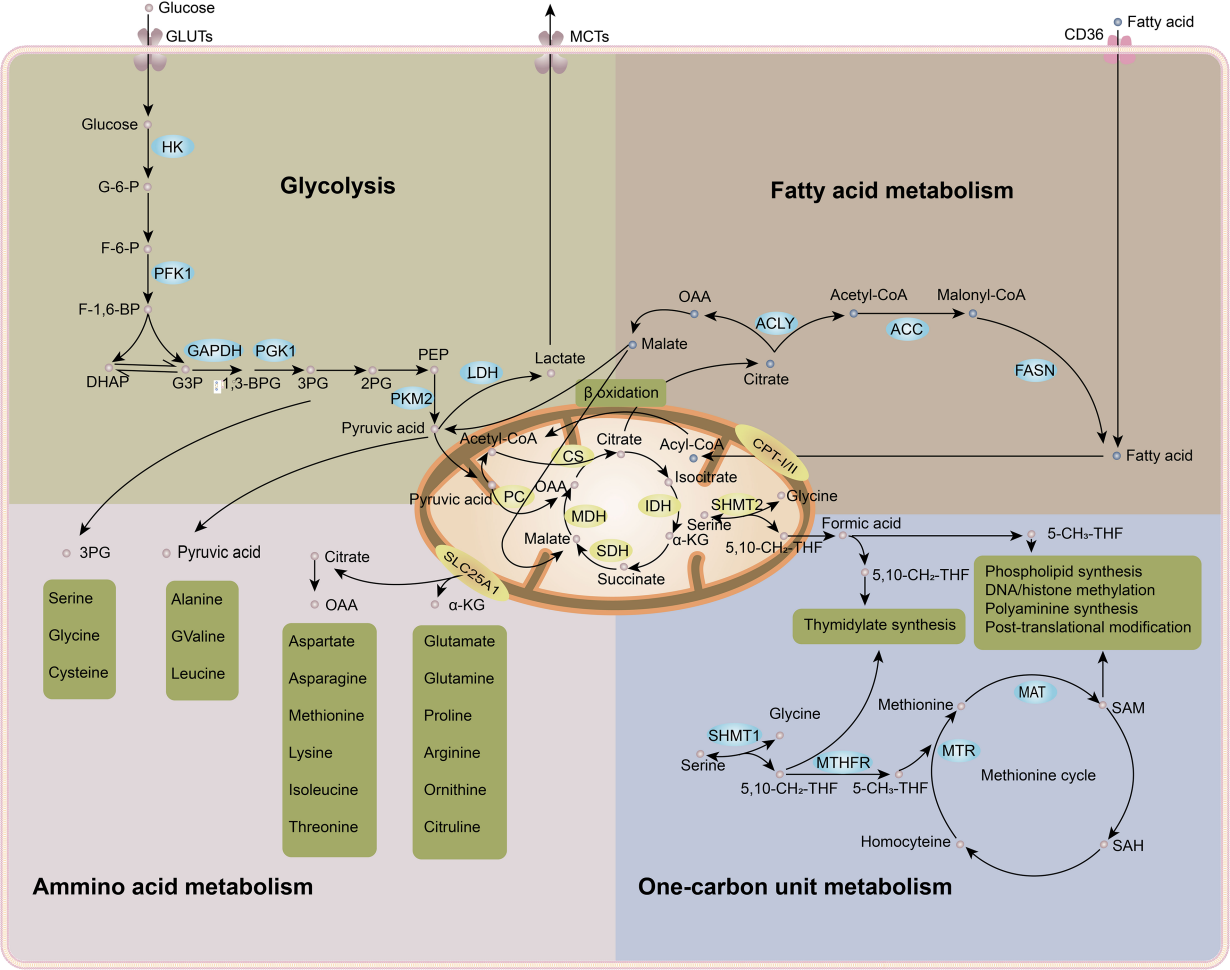
Cellular metabolism is integrally coordinated by mitochondria. Glucose Metabolism: mitochondria serve as the primary site for the aerobic oxidation of glucose; fatty Acid Metabolism: mitochondria host fatty acid β-oxidation, and the acetyl-CoA exported from mitochondria provides a key substrate for de novo fatty acid synthesis; amino acid metabolism: through the carbon skeletons exchange, the mitochondrial TCA cycle contributes to amino acid biosynthesis, while also functioning as the site for oxidative catabolism of amino acid-derived carbon skeletons; one‑carbon units metabolism: mitochondria are a major source of one-carbon units and are essential for maintaining intracellular one‑carbon pool homeostasis.

In summary, mitochondria are not only central to cellular energy supply but also integral to the biosynthesis of proteins, DNA, and lipids through their roles in lipid, amino acid, and one-carbon metabolism, as shown in **Figure 2**. Furthermore, they critically participate in intracellular signal transduction and cellular adaptation by modulating redox homeostasis, the methionine cycle, and folate metabolism.

## 3 Mitochondrial reprogramming in cancer cells

The malignant proliferation of tumor cells is often accompanied by a series of hallmark features: uncontrolled cell division, evasion of growth-inhibitory signals, potent invasive and metastatic capabilities, and sustained angiogenesis (60). Beyond the accumulation of genetic mutations, driving these “malignant behaviors” is a more fundamental alteration in cellular energy metabolism—mitochondrial metabolic reprogramming in tumors (27). This reprogramming in cancer cells encompasses not only the classic Warburg effect but also multi-layered adaptations in the tricarboxylic acid cycle, and mitochondrial respiration, collectively enabling and sustaining the malignant proliferative phenotype.

### 3.1 Warburg effect

The Warburg effect describes the propensity of cells to favor glycolysis and lactate production over oxidative phosphorylation, even in the presence of oxygen (14). First observed by Otto Warburg in the 1920s, this phenomenon laid the foundation for studying mitochondrial metabolism in cancer. Subsequent research, however, has clarified that this is not due to a simple loss of mitochondrial function (27). Instead, tumor mitochondria undergo a sophisticated metabolic reprogramming. This reprogramming serves a dual purpose: it not only enhances glucose uptake and glycolytic flux to meet energy demands but also repurposes mitochondria into biosynthetic hubs (14). Key glycolytic intermediates—such as pyruvate, 3-phosphoglycerate, and acetyl-CoA—are diverted as precursors for lipids, amino acids, and nucleotides. Moreover, the resulting lactate acidifies the tumor microenvironment and contributes to immune evasion (26).

This metabolic shift is orchestrated by oncogenic signaling pathways like PI3K/AKT and Myc, which coordinately upregulate glucose transporters (GLUTs) and key glycolytic enzymes—including HK2, PFK1, and PKM2—to drive the reprogramming process (61). Notably, the regulation of these enzymes exhibits cancer-type specificity and involves multiple layers of control. For instance, in gastric cancer, the acetyltransferase NAT10 stabilizes HK2 mRNA via ac4C modification to promote glycolysis and tumorigenesis (62). In breast cancer, the deubiquitinase OTUB1 stabilizes MYC, which in turn induces HK2 expression and enhances glycolysis (63). Conversely, the immune adaptor STING can suppress HK2 activity, thereby inhibiting glycolysis and promoting anti-tumor immunity (64). In non-small cell lung cancer, constitutive signaling from mutant KRAS or EGFR upregulates PFK1 and PKM2 (65, 66). In gastric cancer, SHP2-mediated dephosphorylation activates PKM2, while ERK1/2 drives its nuclear translocation, both events fueling glycolysis and disease progression (67).

Collectively, these findings extend our understanding beyond the Warburg effect as a mere metabolic curiosity. They establish mitochondrial metabolic reprogramming as a complex, dynamically regulated process underpinned by convergent oncogenic signals and post-translational modifications. This perspective positions metabolic enzymes not merely as passive effectors but as critical signaling nodes that integrate upstream oncogenic cues with downstream biosynthetic and pro-tumorigenic outputs. Such insights reveal novel therapeutic vulnerabilities for targeting cancer metabolism.

### 3.2 TCA cycle in cancer cells

The tricarboxylic acid (TCA) cycle, a central hub for cellular energy metabolism and biosynthesis, undergoes profound reprogramming during tumorigenesis and tumor progression (68, 69). This reprogramming not only fulfills the energetic and biosynthetic demands of rapidly proliferating tumor cells within hypoxic and nutrient-deprived microenvironments, but also drives malignant progression by mediating metabolite-driven epigenetic modifications, activating signaling pathways, and modulating the immune microenvironment (68).

In tumor cells, the activity of pyruvate dehydrogenase (PDH) is inhibited, thereby suppressing the further decarboxylation and oxidation of pyruvate within mitochondria (70). Consequently, pyruvate is diverted to alternative pathways. On one hand, it is converted to lactate via lactate dehydrogenase (LDH), which helps maintain the acidic tumor microenvironment and mediates lactylation modifications (71). On the other hand, pyruvate can be carboxylated to oxaloacetate by pyruvate carboxylase (PC), thereby anaplerotically replenishing TCA cycle intermediates (72). Additionally, tumor cells can generate acetyl-CoA and citrate independently of PDH by utilizing alternative carbon sources such as lactate, acetate, and fatty acids (73, 74).

Furthermore, glutamine contributes to the TCA cycle through glutaminolysis (75). It is deamidated to glutamate, which is subsequently converted to α-ketoglutarate (α-KG) and then to citrate, a process known as glutamine anaplerosis (76). In tumor cells, α-ketoglutarate (α-KG) is not only produced by the decarboxylation of isocitrate catalyzed by isocitrate dehydrogenase (IDH), but is also largely dependent on glutaminolysis and transaminase reactions (77). Beyond its roles in energy and anabolic metabolism, α-KG serves as an essential co-substrate for α-KG-dependent dioxygenases (αKGDDs), which are involved in various tumor-related biological processes (78). For example, prolyl hydroxylase domain (PHD) enzymes, which are αKGDDs, catalyze the hydroxylation of hypoxia-indducible factor-1α (HIF-1α), targeting it for ubiquitin-proteasome degradation (79). Additionally, DNA demethylases such as TET and histone demethylases like JMJD and KDM4A, also αKGDDs, play key roles in epigenetic regulation (80, 81). Notably, gain-of-function mutations in IDH1 or IDH2 genes, frequently observed in gliomas and hematological malignancies, lead to the NADPH-dependent reduction of α-KG to the oncometabolite D-2-hydroxyglutarate (D-2-HG) (82, 83). Structurally similar to α-KG, D-2-HG acts as a competitive inhibitor of αKGDDs. By inhibiting enzymes such as the PHD family, JMJD family, and TET family, D-2-HG impairs cellular epigenetic regulation and hypoxic response, thereby promoting tumorigenesis (82, 84, 85).

Mitochondrial electron transport chain complex II, also known as the succinate dehydrogenase (SDH) complex, catalyzes the conversion of succinate to fumarate within the TCA cycle (86). Deficiencies in succinate dehydrogenase in tumor cells often lead to succinate accumulation. Succinate competitively inhibits αKGDDs by binding to their active sites, which disrupts methylation-dependent regulation and stabilizes HIF-1α under normoxic conditions, thereby contributing to tumor cell stemness and epithelial-mesenchymal transition (EMT) (87, 88). Elevated levels of succinate and its derivative fumarate can inhibit KDM4A and KDM4B, blocking the homologous recombination DNA repair pathway, increasing DNA double-strand breaks, and consequently enhancing sensitivity to PARP inhibitors (89). Moreover, accumulating succinate, particularly in the context of SDH deficiency, promotes epithelial-mesenchymal transition and tumor progression (90, 91). Succinate also modulates tumor cell epigenetics and the function of metabolic enzymes by influencing histone H3 succinylation and the succinylation of proteins such as lactate dehydrogenase A (LDHA) and glutaminase (GLS) (92–94).

In summary, tumor cells shift the TCA cycle away from its canonical role in energy production toward functioning as a biosynthetic and signaling hub. This metabolic rewiring repurposes metabolites such as citrate and α-KG, shifting them from catabolic intermediates to anabolic precursors, and thereby sustains rapid proliferation. Crucially, the resultant accumulation of oncometabolites (D-2-HG, succinate) competitively inhibit αKG-dependent dioxygenases. This enzymatic blockade constitutes a crucial driver mechanism, simultaneously dysregulating epigenetic landscapes (via TET, JMJD inhibition), distorting hypoxic signaling (via PHD inhibition), impairing DNA repair, and promoting angiogenesis, thereby driving tumor initiation, progression, and therapeutic vulnerabilities.

### 3.3 OXPHOS in cancer cells

Although cancer cells predominantly rely on aerobic glycolysis for energy, emerging evidence underscores a complex and critical role for oxidative phosphorylation (OXPHOS) in tumor initiation, progression, therapy resistance, and metastasis (68, 95). Notably, tumor cells that have developed resistance to chemotherapy or targeted therapy, circulating tumor cells with high metastatic potential, and cancer stem cells frequently exhibit a metabolic reprogramming from glycolysis toward enhanced OXPHOS (95–97). In tumors with high metabolic demands, such as leukemia and triple-negative breast cancer, OXPHOS and glycolysis can be synergistically upregulated to fulfill the dual needs of biosynthetic pathways and ATP generation (98, 99). The reprogramming confers a survival advantage to cancer cells in hostile microenvironments, such as during detachment from the primary site or under therapeutic assault (100, 101).

Transcriptional coactivators such as PGC-1α/β are key regulators of mitochondrial biogenesis and OXPHOS, and their expression is upregulated in various drug-resistant and metastatic tumors (102). Resistant cells often exhibit enhanced mitochondrial fusion (e.g., increased expression of MFN1/2), forming an interconnected network that facilitates efficient transfer of energy (ATP) and metabolic intermediates, thereby improving cellular stress tolerance (103–105). The substrates for OXPHOS in tumor cells are not limited to glucose-derived pyruvate. They can flexibly utilize glutamine (supporting the TCA cycle via anaplerosis), fatty acids, and even lactate to fuel mitochondrial respiration (106–109).

The reprogramming of OXPHOS in tumor cells highlights the complexity and adaptability of cancer metabolism. It is not simply the opposite of the Warburg effect but represents a survival strategy adopted by tumors at different developmental stages and under various microenvironmental pressures. Intelligently combining OXPHOS inhibitors with conventional therapies, immunotherapy, and other modalities holds promise as a novel approach to overcoming tumor resistance, preventing metastasis, and improving patient outcomes. The metabolic reprogramming in cancer cells was summarized in **Table 1**.

**Table 1:** Metabolic reprogramming in cancer cells.

Metabolic target	Tumor type	Key mechanism	Significance
HK2	Gastric cancer (62)	NAT10 acetylates HK2 mRNA to activate the glycolytic pathway.	Glucose deprivation induces the degradation of NAT10.
	Breast cancer (63)	OTUB1 deubiquitinate MYC to upregulate HK2 expression.	Targeting the OTUB1-MYC axis disrupts metabolic reprogramming.
	Colon cancer (64)	STING suppresses HK2 activity to inhibits glycolysis.	Targeting the STING-HK2 pathway to reshape cellular metabolism and anti-tumor immunity.
PKM2	Lung cancer (66)	Inhibits PKM2 restores gefitinib sensitivity in EGFR-mutant NSCLC cells.	PKM2/PDK1 dual inhibitor is an effective approach to overcome EGFR-TKI resistance.
	Gastric carcinoma (67)	SHP2 dephosphorylates and activates PKM2 to promote glycolysis.	The SHP2 inhibitor SHP099 blocks SHP2-PKM2 axis and sensitizes tumors to cisplatin.
	Colon cancer (69)	PKM1 mediates OXPHOS dependence and 5-Fu resistance.	OXPHOS inhibitors metformin overcomes 5-FU resistance in colon cancer.
	Colorectal cancer (101)	MYG1 drives colorectal cancer progression via stabilizing PKM2 and activating glycolysis.	MYG1 serves as a diagnostic marker and therapeutic target. in KRAS-mutant colorectal cancer.
PC	Breast cancer (110)	PC upregulates the Wnt/β-catenin/Snail signaling pathway.	ZY-444 inhibits the Wnt/β-catenin/Snail pathway by inactivating PC.
	Liver cancer (111)	PC impaired OXPHOS via ROS-mediated JNK/p38 MAPK pathways.	GAT suppresses liver cancer progression by allosterically activating PC.
	Thyroid cancer (112)	PC activates the AKT/mTOR/SREBP1c pathway to drive fatty acid synthesis and promote cancer progression.	PC–fatty acid synthesis axis is a potential therapeutic target for cancer therapy.
	Ovarian cancer (113)	PC regulates the Wnt/β-catenin signaling pathway to facilitate proliferation and migration of cancer cells.	Erianin exhibits anticancer activity by binding to and inhibiting PC activity.
	Pancreatic cancer (114)	PC downregulates PGC-1α to promote glycolysis and cancer progression.	Inhibiting PC activates the AMPK pathway, and restores tumor sensitivity to metformin.
ASS1	Lung cancer (76)	ASS1 confers ferroptosis resistance and inhibits TCA cycle by promoting reductive carboxylation of GSH.	Targeting ASS1-mTORC1- SCD5 axis sensitizes cells to erastin-induced ferroptosis.
PDH	Osteosarcoma (79)	PHD inhibition by depletion of α-KG induces autophagy and blocks mTORC1 activation by amino acids.	PHDs represent the central integrators of ROS and amino acid signals.
IDH	Glioma (82)	IDH1mut upregulates KRAS and MYC by histone acetylation.	IDH1 serves as a potential target for epigenetic regulation.
	AML (83)	FLT3-ITD activates mutant IDH1 through phosphorylation at Y391 and Y42.	IDH1 and FLT3 represent potential targets in IDH1-mutant AML.
FH/SDH	Renal carcinoma (89)	Accumulating fumarate/succinate inhibits KDM4A/B to impair HRR.	Targeting PARP and FH/SDH exhibits a potential synergistic antitumor mechanism.
α-KGDH	Glioma (92)	α-KGDH interacts with KAT2A to catalyzes histone H3K79 succinylation.	Histone H3K79 succinylation is a potential downstream target in metabolic reprogramming.
LDHA	Thyroid cancer (93)	SIRT5 inhibits LDHA enzymatic activity via desuccinylation at lysine 155.	The lncRNA GLTC-axis is a potential target for cancer metabolic reprogramming.
GLS	Pancreatic cancer (94)	p38 MAPK support tumor growth by enhancing GLS succinylation.	p38 MAPK-GLS axis represents therapeutic targets for pancreatic cancer.
MTCO1	Bladder cancer (95)	MTCO1 upregulates oxidative phosphorylation, thereby driving cancer stemness and cisplatin resistance.	circFOXK2/HSP90β-mediated TCO1 mitochondrial location is a therapeutic target for bladder cancer.
PGC-1α	Breast cancer (96)	PGC-1α promotes oxidative phosphorylation and mitochondrial biogenesis to support tumor progression.	PGC-1α-driven mitochondrial respiration is a key metabolic target in breast cancer
	Colorectal cancer (97)	Exosomal circ_0001610 relieves the suppression of PGC-1α mediated by miR-30e-5p.	Exosomal circ_0001610-miR-30e-5 is a potential target for chemoresistance in colorectal cancer.
Mito-calcium uptake	AML (98)	Inhibiting the mitochondrial calciumuniporter reduces OXPHOS in venetoclax-resistant leukemia stem cells.	Targeting mitochondrial calcium uptake a novel therapeutic strategy to overcome AML drug resistance.
Mitophagy	Lung cancer (100)	PINK1-mediated mitophagy is required for drug resistance in cancer cells.	Combining MAPK inhibitors with mitophagy inhibitors is a novel therapeutic strategy for cancer.
Mitochondrial fusion	Oral squamous cell carcinoma (104)	PA28γ interacts with C1QBP to promote mitochondrial fusion and enhance oxidative phosphorylation.	PA28γ-C1QBP interaction is a key driver and potential therapeutic target in oral squamous cell carcinoma.
	Cervical cancer (105)	Sp1 regulates key genes (Mfn1/2, OPA1, and Drp1) and reprogramming glucose metabolism.	Sp1 is a critical nexus of mitochondrial dynamics and a potential target in cervical cancer.
SLC1A5	Pancreatic cancer (106)	SLC1A5 drives cancer metabolic reprogramming by promoting glutamine import into mitochondria.	HIF-2α/SLC1A5 axis is a new potential target to overcome chemotherapy resistance.

HK2, hexokinase 2; PKM1, pyruvate kinase M1; PKM2, pyruvate kinase M2; PC, pyruvate carboxylase; ASS1, argininosuccinate synthase 1; IDH, isocitrate dehydrogenase; FH, fumarate hydratase; SDH, succinate dehydrogenase; α-KGDH, α-ketoglutarate dehydrogenase; LDH, lactate dehydrogenase; GLS, glutaminase; MTCO1, mitochondrially encoded cytochrome c oxidase I; PGC-1α, peroxisome proliferator-activated receptor gamma coactivator 1-alpha; SLC1A5, solute carrier family 1 member 5; SFRP2, secreted frizzled-related protein 2; NAT10, N-acetyltransferase 10; OTUB1, OTU deubiquitinase 1; MYC, MYC proto-oncogene, bHLH transcription factor; STING, stimulator of interferon genes; EGFR, epidermal growth factor receptor; NSCLC, non-small cell lung cancer; SHP2, Src homology 2 domain-containing phosphatase 2; OXPHOS, oxidative phosphorylation; Fu, 5-fluorouracil; β-catenin, beta-catenin; ROS, reactive oxygen species; JNK, c-Jun N-terminal kinase; p38 MAPK, p38 mitogen-activated protein kinase; AKT, protein kinase B; mTOR, mammalian target of rapamycin; SREBP1c, sterol regulatory element-binding protein 1c; TCA, tricarboxylic acid cycle; GSH, glutathione; PHD, prolyl hydroxylase domain; mTORC1, mammalian target of rapamycin complex 1; IDH1mut, mutated isocitrate dehydrogenase 1; FLT3-ITD, Fms-like tyrosine kinase 3 – internal tandem duplication; KRAS, Kirsten rat sarcoma viral oncogene homolog; KDM4A/B, lysine demethylase 4A/B; HRR, homologous recombination repair; KAT2A, lysine acetyltransferase 2A; H3K79, histone H3 lysine 79; SIRT5, sirtuin 5; LDHA, lactate dehydrogenase A; PINK1, PTEN-induced putative kinase 1; MYG1, MYG1 exonuclease and mitochondrial assembly factor; PA28γm, proteasome activator 28γ; C1QBP, complement component 1 Q binding protein; Sp1, specificity protein 1; Mfn1/2, mitofusin 1/2; OPA1, optic atrophy 1; Drp1, dynamin-related protein 1; NFAT, nuclear factor of activated T cells; TOX, thymocyte selection-associated high mobility group box protein; TDP-43, TAR DNA-binding protein 43; mtDNA, mitochondrial DNA; VDAC2, voltage-dependent anion channel 2; IFN, interferon; PDK1, pyruvate dehydrogenase kinase 1; TKI, tyrosine kinase inhibitor; AMPK, adenosine monophosphate-activated protein kinase; SREBP1, sterol regulatory element-binding protein 1; SCD5, stearoyl-CoA desaturase 5; GAT, gamma-aminobutyric acid transporter; AML, acute myeloid leukemia; PARP, poly(ADP-ribose) polymerase; lncRNA, long non-coding RNA; SUCLA2, succinate-CoA ligase ADP-forming subunit beta; HSP90β, heat shock protein 90β; HIF-2α, hypoxia-inducible factor 2 alpha.

## 4 Mitochondrial reprogramming in immune cells

Tumor cells, through metabolic reprogramming, competitively uptake nutrients and accumulate metabolic waste, which in turn influences the metabolic remodeling of immune cells, collectively establishing a highly immunosuppressive metabolic microenvironment (23, 24, 27). Within this context, the functional and dynamic remodeling of mitochondria plays a central role. Understanding mitochondrial reprogramming in immune cells is essential to uncovering the mechanisms by which tumors metabolically suppress immune function, and how therapeutic interventions can restore or enhance the metabolic activity of immune cells to promote antitumor immunity.

### 4.1 T cells

T cells in the tumor microenvironment encompass heterogeneous subpopulations with distinct metabolic and mitochondrial profiles (115, 116). Naïve T cells rely on oxidative phosphorylation (OXPHOS) and fatty acid oxidation (FAO) to maintain quiescence and are characterized by small, fragmented mitochondria (117). Upon activation, effector T cells undergo metabolic reprogramming toward aerobic glycolysis and Drp1-mediated mitochondrial fission, which supports rapid clonal expansion and effector functions (118). A subset of these cells differentiate into memory T cells, which harbor fused, elongated mitochondria with enhanced spare respiratory capacity (SRC) and rely on FAO for long-term persistence (119, 120). In contrast to memory T cells, under chronic antigen stimulation such as the tumor microenvironment, CD8+ T cells progressively differentiate into exhausted subsets: progenitor exhausted (Tpex) cells (TCF1^+^ CXCR5^+^ PD-1*^int^*) retain mitochondrial function and respond to immune checkpoint blockade (ICB), whereas terminally exhausted (Ttex) cells (PD-1*^high^* TIM-3^+^) exhibit severe mitochondrial dysfunction, fragmented and depolarized mitochondria, and impaired OXPHOS (18, 21, 121, 122).

Mitochondrial metabolism determines T cell function and differentiation through dynamic quality control mechanisms, including biogenesis, fission/fusion dynamics, mitophagy, and intercellular mitochondrial transfer (123). Mitochondrial biogenesis is tightly coupled to T cell activation. Upon TCR engagement, calcium influx activates the Ncoa2-CREB pathway, which binds to enhancer regions of Ppargc1a (encoding PGC-1α) to drive its transcription (124). PGC-1α serves as the master regulator of mitochondrial biogenesis, coordinating the expression of nuclear-encoded mitochondrial genes (125). Beyond ATP production, mitochondria-derived metabolites such as citrate and α-ketoglutarate (α-KG) regulate T cell differentiation by respectively promoting histone acetylation (e.g., at the Ifng locus) and serving as demethylase cofactors (126–128). Mitochondrial dynamics critically influence T cell fate decisions. In effector T cells, PI3K-Akt-mTOR signaling promotes Drp1-mediated mitochondrial fission, which supports rapid proliferation and effector functions (118). Conversely, Drp1 inhibition or deletion shifts T cells toward a memory phenotype and enhances antitumor immunity (129), positioning mitochondrial fission as a therapeutic target for promoting durable T cell responses. Mitophagy, the selective clearance of damaged mitochondria via the PINK1-Parkin pathway, maintains mitochondrial integrity (100). In the tumor microenvironment, chronic antigen stimulation drives USP30 upregulation, which suppresses PINK1/Parkin-mediated mitophagy, leading to accumulation of dysfunctional mitochondria, excessive ROS production, and ultimately loss of T cell effector function (130). Additionally, tumor cells transfer mitochondria harboring mtDNA mutations to effector T cells via tunneling nanotubes (TNTs) and extracellular vesicles (EVs). Importantly, these tumor-derived mitochondria carry the mitophagy inhibitor USP30 on their surface, enabling them to evade mitophagic clearance within T cells, thereby driving effector T cell dysfunction and immune evasion (23).

### 4.2 B cells

In the tumor microenvironment (TME), the anti−tumor capacity of B cells is closely associated with their spatial organization, which exhibits three distinct patterns: lymphoid aggregates, immature tertiary lymphoid structures (TLS), and mature TLS (131–133). Unlike the other two patterns, mature TLS possess germinal center (GC)-like structures analogous to those found in lymph nodes. Within these structures, germinal center B cells undergo robust proliferation, activation, affinity maturation, and class switch recombination to produce high-affinity anti-tumor antibodies, thereby promoting effective anti-tumor immunity and serving as important prognostic biomarkers for immunotherapy response (134, 135). The mitochondria of GC B cells are highly dynamic, with significantly upregulated transcription and translation rates associated with the activity of transcription factor A, mitochondrial (TFAM). Loss of TFAM impairs the chemokine−guided motility of GC B cells, leading to their spatial disorganization and subsequent inhibition of TLS maturation (136). Although mitochondria and oxidative phosphorylation (OXPHOS) are essential for GC B cell motility and TLS maturation, activated B cells entering the GC undergo a metabolic switch toward glycolysis. This switch supports centroblast proliferation, affinity maturation, and class switch recombination by enabling MCT1−dependent pyruvate import into the nucleus, where pyruvate promotes H3K27 acetylation and drives AID transcription (137).

B cells predominantly exert their immunomodulatory roles outside mature TLS (138, 139). Their substantial consumption of nutrients such as glucose and glutamine within the TME can create competition with T cells, potentially indirectly compromising T cell function (12). Furthermore, specific metabolic preferences can program the immunoregulatory functions of B cells. For example, in colorectal cancer, immunoregulatory B cells expressing mitochondrial leucyl-tRNA synthetase 2 (LARS2) preferentially uptake leucine to drive mitochondrial NAD^+^ regeneration and oxidative metabolism (140). This metabolic program enhances the production of TGF-β1, which in turn suppresses anti-tumor immunity and promotes immune evasion. Targeting this metabolic vulnerability, studies suggest that leucine restriction strategies could inhibit the function of this B cell subset.

### 4.3 NK cells

Natural killer (NK) cells are critical innate immune effectors within the tumor microenvironment (TME). Their activation initiates from a dual-receptor logic: activating receptors such as NKG2D and DNAM-1 recognize stress-induced ligands on tumor cells, while the frequent downregulation of MHC class I on tumors releases inhibitory signaling mediated by KIRs and NKG2A, collectively triggering NK cell activation (141–143). Upon activation, NK cells undergo tightly regulated metabolic reprogramming to support effector functions. This metabolic shift follows a sequential program: an initial rapid induction of aerobic glycolysis, driven by TNFα/AKT/mTORC1 signaling, provides immediate biosynthetic precursors and energy (144); subsequently, NK cells transition to oxidative phosphorylation (OXPHOS) as the dominant metabolic pathway for sustained effector activity, including cytokine production and tumor killing (145).

Within the tumor microenvironment, inhibitory factors such as lactate, acetoacetic acid, together with tumor−expressed surface proteins including CD155, PD−L1, and TRAIL, impair NK cell function by inducing mitochondrial fragmentation and lactate−driven post−translational modifications (146–149). These alterations lead to mitochondrial dysfunction and reduce the secretion of perforin, granzymes, and cytokines, thereby diminishing tumor−killing capacity. Furthermore, the inhibitory transcription factor FOXP3 reprograms NK cell metabolism, shifting it from glycolysis toward oxidative phosphorylation, a metabolic shift associated with the acquisition of an immunosuppressive phenotype (150).

### 4.4 Dendritic cells

Dendritic cells (DCs) form a critical bridge between innate and adaptive immunity, with their function intrinsically linked to metabolic reprogramming. Among subsets, cDC1s are specialized in priming CD8^+^ T cell responses via MHC class I presentation, while cDC2s engage CD4^+^ T cells through MHC class II (151). Additional subsets like pDCs and LAMP3^+^ DCs contribute distinct cytokine profiles, shaping the broader immune landscape and serving as potential immunotherapy biomarkers (151, 152).

Dendritic cells (DCs) highly express monocarboxylate transporter 1 (MCT1), enabling them to actively take up lactate from the tumor microenvironment (153). The high-lactate environment resulting from the tumor Warburg effect leads to intracellular lactate accumulation in DCs, causing metabolic and functional impairment, which subsequently suppresses their antigen presentation and immunostimulatory capacity (154, 155). Blocking the lactate transport pathway enhances oxidative phosphorylation in DCs. Notably, the increase in the metabolite alpha-ketoglutarate (α-KG) modulates DNA methylation, thereby boosting the anti-tumor function of DCs (156).

Furthermore, mitochondrial function plays a crucial regulatory role in determining the immunophenotype of DCs. Studies show that DCs with healthy mitochondria, upon activation of the cGAS-STING pathway, exhibit enhanced anti-tumor immunity (157, 158). Conversely, in DCs with damaged mitochondria, cGAS-STING pathway activation induces PD-L1 expression, consequently suppressing anti-tumor immune responses (159, 160). Collectively, future immunotherapy strategies may focus on reversing tumor microenvironment-induced suppression of dendritic cells by targeting lactate metabolism and restoring mitochondrial function, thereby enhancing their antigen-presenting capacity and reshaping anti-tumor immune responses. The metabolic reprogramming in immune cells was summarized in **Table 2**.

**Table 2:** Metabolic reprogramming in immune cells.

Immune cells	Metabolic targets	Key mechanism	Significance
T cells	HIF-1α (18)	Mitochondrial dysfunction and HIF-1α–driven glycolysis promotes T cells exhaustion.	Mitochondrial insufficiency and HIF-1α-glycolysis axis are the potential targets sustain stemness of T cells.
	OXPHOS (16)	Persistent antigen impairs OXPHOS to limited self-renewal of CD8+ T cells.	Preserving mitochondrial redox balance boosts stemness of T cells and anti-tumor immunity.
	Pyruvate (15)	IL-10 maintains stemness T cells depending on pyruvate importing into mitochondria.	IL-10 restores proliferative capacity of TIL, synergizing with ACT.
	PKM2 (161)	PKM2 activation boosts T cell effector function via one-carbon and mitochondrial reprogramming.	PKM2 serves as a target to enhance T cell fitness and synergize with checkpoint blockade.
	LARP4 (162)	LARP4 drives T cell exhaustion by hypertranslating OXPHOS subunits.	LARP4-driven translational dysregulation as a driver of T cell exhaustion and a target for AC.
	SFRP2 (163)	SFRP2 m1A RNA modification activates the NFAT/TOX signaling axis, thereby driving T-cell exhaustion.	SFRP2-NFAT/TOX axis as a novel mechanism and potential target of immune evasion.
B cells	TFAM (136)	TFAM-mediated mitochondrial -translation promotes B cells development and GC entry.	TFAM and mitochondrial -translation serve as direct targets to control B cell fate.
	OXPHOS (164)	Acar enhanced the activity of mitochondrial ETC by acetylation of H3K27.	Targeting Erbin in platelets promotes B cell-mediated antitumor immunity.
	GLUT1 (17)	GLUT1 fuels GC B cells to sustain antibodies without glucose.	This work establishes glucose demand for humoral immunity and reveals B cell hexose flexibility.
	OXPHOS (137)	MCT1-mediated pyruvate metabolism is required for IgG antibody CSR through H3K27 acetylation.	MCT1 is a metabolic-epigenetic regulator for vaccination and autoimmunity.
	Glycolysis (12)	Tumor-associated B cells exhibits high glycolytic and OXPHOS activities, thereby suppress T cell function.	B cells represents a therapeutic target for immune evasion.
NK cells	TNFR2 (144)	NK proliferation and antiviral function depends on inducing aerobic glycolysis.	TNFα/TNFR2 axis is a key link between inflammation and NK metabolism, regulating innate immune expansion.
	NAD metabolism (145)	NK cells enhances the killing function via NAD metabolism during metabolic disturbances.	Metabolic resilience of NK cell can synergize with tumor immunotherapy.
	Lactate (149)	ROCK1 lactylation induces mitochondrial fission and impairs NK cytotoxicity.	SIRT3 activation reverses lactate-mediated ROCK1 lactylation, as a target to enhance NK immunotherapy.
	Drp1 (147)	mTOR-Drp1 drives NK mitochondrial hyperfission to impair function and survival.	Hypoxia-induced mitochondrial fission as a target for immune escape.
	LDHB (150)	The immunosuppression of FOXP3^+^ NK/T cells relies on MCT1/LDHB induced glycolysis and histone lactylation.	MCT1 holds potential to reverse cold tumors to hot ones.
Dendritic cells	OXPHOS (155)	MerTK^+^ DC exhibits elevated mitochondrial respiration and reduced T-cell stimulatory capacity.	Mitochondria in MerTK^+^ DC is a metabolic target to overcome checkpoint resistance.
	TFAM (157)	mtDNA in DC activates cGAS-STING to enhance presentation.	TFAM inducing mtDNA leakage as a metabolic target for immunotherapy.

HIF-1α, hypoxia-inducible factor 1-alpha; ROS, reactive oxygen species; OXPHOS, oxidative phosphorylation; PKM2, pyruvate kinase M2; SFRP2, secreted frizzled-related protein 2; TFAM, mitochondrial transcription factor A; GLUT1, glucose transporter 1; TNFR2, tumor necrosis factor receptor 2; Drp1, dynamin-related protein 1; LDH, lactate dehydrogenase; MerTK, MER proto-oncogene tyrosine kinase; IL-10, interleukin-10; LARP4, La-related protein 4; NFAT, nuclear factor of activated T cells; TOX, thymocyte selection-associated high mobility group box protein; ETC, electron transport chain; PPP, pentose phosphate pathway; MCT1, monocarboxylate transporter 1; IgG, immunoglobulin G; CSR, class-switch recombination; GC, germinal center; ROCK1, Rho-associated protein kinase 1; mTOR, mammalian target of rapamycin; LDHB, lactate dehydrogenase B; FOXP3, forkhead box P3; DC, dendritic cell; mtDNA, mitochondrial DNA; cGAS, cyclic GMP-AMP synthase; STING, stimulator of interferon genes; CD8, cluster of differentiation 8; TIL, tumor-infiltrating lymphocytes; ACT, adoptive cell transfer; TNFα, tumor necrosis factor-alpha; SIRT3, sirtuin 3.

## 5 Mitochondria: novel strategies for cancer immunotherapy

Mitochondria, the central hubs of cellular energy production and key regulators of apoptosis and signaling, have emerged as compelling therapeutic targets in oncology, particularly within the field of immunotherapy (27). The efficacy of conventional treatments is often limited by the immunosuppressive tumor microenvironment (TME) and the functional exhaustion of effector T cells (14, 15). In recent years, strategies aimed at modulating mitochondrial function have opened new dimensions for overcoming these challenges, focusing on direct tumor destabilization, remodeling of the immunometabolic landscape, and potentiation of immune cell efficacy.

### 5.1 Targeting mitochondria in cancer cells

While aerobic glycolysis serves as the primary source of ATP in tumor cells, mitochondrial function remains essential for their survival (14). Meanwhile, cancer cells can acquire functional mitochondria from immune cells to enhance the metabolic plasticity and survival capacity (165). Therefore, mitochondria represent a metabolic vulnerability and a highly promising therapeutic target in cancer. Therapeutic targeting of serine metabolism, the mitochondrial outer membrane channel protein VDAC2, and the DNA/RNA-binding protein TDP43, can trigger the release of mitochondrial DNA, thereby activating the cGAS-STING pathway (158, 166, 167). This cascade subsequently induces immunogenic cell death in tumor cells and stimulates an anti-tumor immune response.

Specific mitochondrial-targeted agents, such as Mito-FFA, mitoFePDA@R nanoparticles, as well as mitochondria-targeted photodynamic therapy, induce the release of damage-associated molecular patterns (DAMPs), including IL-1β, IL-18, HMGB1, and ATP, from tumor cells (168–170). This process promotes dendritic cell maturation and antigen presentation, enhances the infiltration of cytotoxic T lymphocytes, and converts “cold” tumor to a “hot” one. However, mitochondria play heterogeneous roles across different cell types within the tumor microenvironment. Mitochondrial damage in tumor cells is associated with immunogenic cell death and enhanced anti-tumor immunity (158, 166, 167, 169). Conversely, mitochondrial dysfunction in effector immune cells, such as T cells and B cells, is linked to cellular senescence, exhaustion, and impaired anti-tumor immunity (16, 21, 167, 171). Therefore, developing mitochondrial-targeted therapies with greater specificity for tumor cells represents a pressing clinical imperative.

### 5.2 Targeting mitochondria in immune cells

As previously discussed, mitochondria play critical roles in determining the immunoregulatory functions of immune cells within the tumor microenvironment (TME). Therefore, targeting the mitochondria of these cells represents a promising strategy to enhance anti-tumor immunity. Palmitate in the TME was shown to irreversibly impair CTL mitochondrial metabolism, reducing histone acetylation and chromatin accessibility, and suppressing T cell replication and effector gene transcription (172). SPHK2 was identified as a key mediator of this process, and its pharmacological inhibition restored mitochondrial fitness and anti-tumor activity. B cells can promote anti-tumor immunity through mitochondrial-dependent mechanisms. Erbin-deficient platelets increase mitochondrial OXPHOS and secrete acyl-carnitine, which enhances mitochondrial ETC complex activity and OXPHOS in B cells via H3K27 acetylation, thereby promoting B cell-mediated anti-tumor immunity against colorectal cancer metastasis (164).

Metabolic stresses in the tumor microenvironment (TME) progressively impair NK cells, potent cytotoxic lymphocytes, as evidenced by their reduced mitochondrial mass and fragmented morphology (173). CRISPR-Cas9-mediated DRP1 knockout protects NK cells from hypoxia-induced dysfunction by preserving mitochondrial integrity, a prerequisite for their cytotoxic function (174). OPA1-NRF1-driven mitochondrial fitness in cDC1s controls antigen processing and co-stimulation for anti-tumor immunity; its decline during tumor progression can be rescued by intratumoral delivery of mitochondria-polarized cDC1s, which boosts immune checkpoint blockade therapy (175). Mitochondrial ATP production is a critical bottleneck for cGAMP synthesis and STING activation in DCs, and manganese nanoadjuvants relieve this bottleneck by normalizing ETC function to boost ATP production, serving as potent immune adjuvants (176).

### 5.3 Targeting mitochondria in CAR-T

Tumor cells exploit the Warburg effect to generate an acidic microenvironment, which drives T cell exhaustion and confers resistance to both tumor-infiltrating and adoptively transferred CAR-T cells (10, 21). This mechanism constitutes a major obstacle to the efficacy of CAR-T therapy in solid tumors, with mitochondrial dysfunction being a central inducer of this dysfunctional state (16, 18, 26). Consequently, ex vivo engineering of CAR-T cells has emerged as a prominent research focus for improving their performance against solid tumors. Key strategies include activating transcription factors that suppress exhaustion—such as FOXO1, KLF2, TCF7, and BCL6—while inhibiting pro-exhaustion factors like TOX/TOX2, NR4A, NFAT, and BLIMP1 (6, 8, 121, 163, 177–179). Preclinical studies indicate that these interventions aimed at mitigating T cell exhaustion and restoring mitochondrial function can enhance efficacy of cancer immunotherapy.

Meanwhile, metabolic interventions including PKM2 agonism, metformin treatment, and DCA conditioning can enhance mitochondrial biogenesis and function in T cells, improving effector functions, memory phenotype, and stemness for adoptive cell therapy (161, 180, 181). Furthermore, promoting mitochondrial redox homeostasis, maintaining protein folding homeostasis, and preventing the transfer of damaged mitochondria from tumor cells to T cells represent critical future directions for optimizing CAR-T therapy (38, 162, 182). Nonetheless, these approaches carry significant clinical risks. Sustained T cell activation is associated with a heightened incidence of cytokine release syndrome (CRS) (183). Moreover, the forced overexpression of stemness-associated transcription factors, coupled with the random genomic integration of transgenes, may potentially increase the risk of T cell malignant transformation.

## 6 Future perspectives: emerging fields in mitochondrial therapeutics

The rapidly evolving field of mitochondrial therapeutics is advancing past traditional strategies centered on small molecules and cellular genetic engineering. A number of innovative approaches, supported by recent technological advances, are poised to significantly enhance the targeting accuracy and therapeutic potential of cancer immunotherapy.

### 6.1 Mitochondria as a cancer vaccine platform

Traditional antitumor vaccines face inherent limitations in clinical efficacy, primarily attributed to their weak immunogenicity and the substantial heterogeneity observed within tumor tissues (184). These constraints markedly impair the induction of potent and sustained antitumor immune responses. In this context, the mitochondrial platform-based cancer vaccine has emerged as a highly promising and innovative therapeutic strategy that addresses several critical limitations of conventional approaches. Mitochondria serve as a rich repository of endogenous immune activators, collectively termed damage-associated molecular patterns (DAMPs), including key components such as mitochondrial DNA (mtDNA), cardiolipin, adenosine triphosphate (ATP), and reactive oxygen species (ROS) (185). Notably, these molecules represent some of the most potent endogenous immunostimulants identified to date. They are capable of potently activating dendritic cells (DCs) and triggering key immune signaling cascades, including the cGAS-STING pathway and TLR9-mediated signaling, thereby facilitating the formation of long-lasting memory T cell responses (186, 187).

Wei and colleagues recently reported that engineered mitochondria can serve as a novel cancer vaccine platform, demonstrating antigen-specific T cell responses and tumor protection in murine models (188). Specifically, they engineered mitochondria loaded with tumor-associated antigens, designated as OVA-MITO and TRP2-MITO, to function as next-generation cancer vaccines. These engineered mitochondrial vaccines exhibited extraordinary capacity to recruit and activate dendritic cells at the site of administration. This targeted engagement of local DCs enabled efficient priming of the adaptive immune system, ultimately culminating in the induction of robust, tumor-specific cellular immunity.

Collectively, these findings underscore the transformative potential of mitochondrial platform-based vaccines as a compelling frontier in cancer immunotherapy. The unique combination of intrinsic DAMP-mediated immunostimulation and customizable antigen loading positions this platform as a versatile and potent approach for overcoming the limitations of conventional tumor vaccines. Future research directions should focus on optimizing mitochondrial engineering techniques, exploring combination strategies with existing immunotherapies such as immune checkpoint inhibitors, and advancing clinical translation to fully realize the therapeutic potential of this innovative platform.

### 6.2 Precision mitochondrial genome editing

Mitochondrial gene editing represents a novel strategy for precision regulation of the cellular “powerhouse,” addressing key challenges in oncology such as treatment resistance and immune evasion. However, the inability of single-guide RNA (sgRNA) to freely enter mitochondria prevents the CRISPR-Cas9 system from achieving precise mitochondrial DNA (mtDNA) editing (189). To overcome this limitation, researchers have engineered TALE-linked deaminases (TALEDs). This innovative approach utilizes transcription activator-like effector (TALE) proteins for specific recognition of target sequences within mtDNA, coupled with deaminase domains to enable targeted base conversion (190, 191). Li et al. successfully developed a high-precision mitochondrial DNA cytosine base editor (DdCBE–TOD) using an artificial intelligence-assisted *de novo* protein design strategy, enabling single-base-resolution precision editing and significantly improving the accuracy of mitochondrial DNA editing (192). Yin et al. screened and identified a 40-nucleotide aptamer (IM83) that effectively delivers sgRNA into the mitochondrial matrix. By employing a mitochondrially targeted adenine base editor, they achieved, for the first time, CRISPR-based editing of mtDNA (193). Jiang and colleagues successfully achieved functional complementation of mutant mtDNA by utilizing fluorinated lipid nanoparticles conjugated with a mitochondrial targeting sequence (M) for mitochondria-targeted gene delivery (194).

### 6.3 Engineered mitochondrial transplantation and transfer

Given that mitochondrial dysfunction and metabolic reprogramming underlie the immunosuppressive phenotype of immune cells in the TME, restoring metabolic fitness through mitochondrial replacement represents a transformative strategy to boost cancer immunotherapy efficacy (195, 196). However, key technical hurdles remain to be addressed: sourcing high-quality donor mitochondria and developing efficient delivery systems for their uptake by target immune cells (197). By activating the CD38/IP3R/Ca²^+^ signaling axis in MSCs, Wang et al. engineered them to produce mitochondria-enriched extracellular vesicles that efficiently delivered functional mitochondria to recipient cells and restored cellular metabolism (195). This work provides a platform for vesicle-mediated mitochondrial transfer in immunotherapy.

Intercellular nanotubular structures enable bone marrow stromal cells (BMSCs) to deliver mitochondria precisely to CD8+ T cells, a process mechanistically dependent on the Talin 2 protein (196). Therefore, CAR-T cells could be engineered to overexpress Talin 2 and periodically receive exogenous mitochondrial supplements. This strategy is proposed to sustain their metabolic fitness and thereby enhance persistent anti-tumor efficacy.

To develop more precise mitochondrial delivery strategies, Baldwin et al. elucidated a contact-dependent mechanism for intercellular mitochondrial transfer (196). They identified that BMSCs transfer mitochondria to CD8^+^ T cells via Talin-2-dependent nanotubular structures. Mitochondria-acquired (Mito^+^) CD8^+^ T cells exhibited enhanced respiratory capacity, reduced exhaustion, and improved tumor infiltration, leading to superior antitumor efficacy in melanoma and leukemia models. In future, the convergence of vesicle engineering and nanotubular biology positions engineered mitochondrial transfer as a next-generation modality to substantially enhance the durability and effectiveness of cancer immunotherapies, including CAR-T and immune checkpoint blockade.

## 7 Limitations

Several limitations should be acknowledged. First, the field of mitochondrial transfer is still nascent, and most evidence derives from murine models or *in vitro* experiments; clinical validation remains lacking. Second, the molecular mechanisms governing intercellular mitochondrial transfer—particularly the regulation of nanotube formation and vesicle-mediated delivery—are not fully elucidated and likely vary across tumor types and immune subsets. Third, the safety and immunogenicity of exogenous mitochondrial transplantation in humans have not been systematically evaluated. Addressing these gaps will be essential for translating these strategies into clinical applications.

## 8 Conclusion

In summary, mitochondria have evolved beyond their traditional role as mere cellular powerhouses to function as central integrators of metabolism and critical regulators of antitumor immunity. Within the tumor microenvironment, mitochondrial reprogramming exhibits a dual nature: in cancer cells, it sustains proliferation, promotes anabolic biosynthesis, and drives immune evasion; in immune cells, it dictates functional fate, with metabolic fitness being indispensable for effector potency, memory formation, and resistance to exhaustion.

Therapeutic strategies emerging from this understanding are advancing along two synergistic fronts. The first targets tumor mitochondria as a critical vulnerability, aiming to induce immunogenic cell death, activate innate immune sensing pathways such as cGAS-STING, and thereby reverse the immunosuppressive “cold” tumor state. The second focuses on augmenting mitochondrial function in immune cells through genetic and metabolic engineering—for example, in CAR-T cell therapies—to enhance their persistence and resilience. However, the inherent heterogeneity of mitochondrial function across different cell types underscores the necessity for cell-type-specific targeting, essential to prevent inadvertent suppression of antitumor immunity while attacking malignant cells.

Looking ahead, advances such as mitochondrial vaccines, precise mtDNA editing, and engineered mitochondrial transplantation herald a future in which this organelle can be therapeutically modulated with unprecedented specificity and control. Translating these sophisticated paradigms from promising preclinical concepts into safe and effective clinical modalities remains the paramount challenge. Success in this endeavor will undoubtedly represent a significant advance in the ongoing effort to harness the immune system for cancer therapy.
